# Supranuclear Palsy as an Initial Presentation of the Adult-Onset Niemann-Pick Type C

**DOI:** 10.3390/neurolint16030042

**Published:** 2024-05-13

**Authors:** Ali A. Mohamed, Willy Gan, Denis Babici, Veronica Hagan, Raphael Wald, Marc Swerdloff

**Affiliations:** 1 Charles E. Schmidt College of Medicine, Florida Atlantic University, Boca Raton, FL 33431, USA; 2 Department of Neurology, Florida Atlantic University Charles E. Schmidt College of Medicine, Boca Raton, FL 33431, USA

**Keywords:** vertical supranuclear gaze palsy, round the house, sea-blue histiocytes, vorinostat, miglustat, 2-hydroxypropyl-β-cyclodextrin

## Abstract

(1) Background: Niemann–Pick type C1 (NP-C1) is a lysosomal storage disorder that results in the defective trafficking of cholesterol and other cellular lipids in the endosomal–lysosomal pathway. This rare autosomal recessive disorder presents in three forms based on the age of onset. The adult form presents in patients greater than 15 years of age but is rarely seen after the age of 30. Common symptoms of the late adult-onset category of NP-C1 include progressive cognitive impairment and ataxia, with psychiatric and movement disorders presenting less frequently than in other forms of NP-C1. Dystonic movement disorders present most frequently, along with chorea, myoclonus, and parkinsonism. Herein, we present a rare case of NP-C1, diagnosed at age 35 with an initial symptom of supranuclear palsy. The goal of the presented case is to highlight the importance of the neurological examination and an inclusive differential diagnosis in patients with new-onset supranuclear palsy. (2) Methods: A single case report. (3) Results: A 46-year-old male with a past medical history of NP-C1 was admitted to the hospital for respiratory distress. He was noted to have a supranuclear gaze palsy with partially preserved voluntary saccades to the right. His mother revealed that he first had difficulty moving his eyes at the age of 34. After multiple consultations and genetic testing one year later, he was diagnosed with NP-C1. (4) Conclusions: Because NP-C1 affects many regions of the brain responsible for eye movements, neurological eye assessments can be a useful tool in diagnoses. Furthermore, eye movement abnormalities may be the initial presenting symptom of NP-C1, predisposing patients to misdiagnosis with progressive supranuclear palsy and other conditions that may mimic early-stage NP-C1. Definitive diagnosis is achieved through genetic testing. Filipin staining test was the gold standard in the past. The NP-C Suspicion Index was developed to assist in diagnoses, but its efficacy is unclear with late adult-onset NP-C1. Although no cure exists, early identification can facilitate an improved symptom management course for patients. Miglustat, a glucosylceramide synthase (GCS) inhibitor, is the approved therapy in Europe specific to NP-C1 for slowing and preventing the neurological manifestations of NP-C1. Delays between symptom onset and treatment initiation are likely to result in poorer outcomes and a progression of neurological symptoms. High doses may present tolerance concerns, especially in cases of delayed treatment and advanced neurological deficit.

## 1. Introduction

Niemann–Pick type C (NP-C) is a rare autosomal recessive lysosomal storage disorder which occurs due to an *NPC1* or *NPC2* gene mutation [1,2]. This results in the defective trafficking of cholesterol and other cellular lipids in the endosomal–lysosomal pathway. NP-C presents as an infantile, juvenile, or adult form based on the age of onset. An early infantile form has an age of onset of less than 2 years old, is neurovisceral, and usually presents with hepatosplenomegaly, neonatal jaundice, delayed developmental motor milestones, and hypotonia. The juvenile form, from age 6 to 15 years old, presents with cognitive impairment, progressive ataxia with frequent falls, dystonia, and vertical supranuclear gaze palsy (VSGP). The adult form presents in patients greater than 15 years of age and clinically resembles the juvenile form. Very late adult-onset NP-C, defined as diagnosis after age 30, is rare [3]. Progressive cognitive impairment with ataxia is the most common clinical feature of very late adult-onset NP-C. Psychiatric and movement disorder presentations are also seen in very late adult-onset NP-C but less frequently. Dystonic movement disorders are the most frequent in very late adult-onset NP-C, followed by chorea, myoclonus, or parkinsonism [3]. Parkinsonism in this patient group is typically mild, consisting of bradykinesia, hypomimia, axial rigidity, or an isolated resting tremor. Gelastic cataplexy, a conscious episode of a loss of muscle tone triggered by laughing, is unique to NP-C, present in 16% of patients. VSGP presents in 65% of patients [4].

NP-C affects regions of the brain responsible for eye movements, including the cerebral cortex, cerebellum, brainstem, thalamus, and basal ganglia [5]. Neurological eye assessments can therefore be a powerful diagnostic tool, especially in cases where eye movement abnormalities may be the initial presenting symptom of NP-C. A high level of clinical suspicion is crucial in these cases as patients may be misdiagnosed with progressive supranuclear palsy (PSP) and other conditions that may mimic early-stage NP-C. Herein, we present a patient with late adult-onset NP-C, diagnosed at age 35, with eye movement abnormalities identified as supranuclear gaze palsy on neurological eye exam.

## 2. Case Presentation

A 46-year-old male with known NP-C1 diagnosed 10 years ago presented to our hospital with shortness of breath and a cough and was diagnosed with pneumonia and a cardiac arrythmia (Brugada syndrome type 1A) following cardiac evaluation. He had been treated with miglustat since the initial diagnosis. Neurologically, he was cognitively impaired, oriented only to his name. He intermittently followed simple commands that did not require him to cross the midline and had hypophonic dysarthric speech that was hard to understand. To command, his saccades were absent to the left and upward, and limited to the right. This inability was easily overcome with oculocephalic head movements in all directions (Video S1). He had clumsy hand movements with increased arm tone bilaterally and his patellar reflexes were brisk bilaterally. At baseline, the patient was unable to walk independently and had been using a wheelchair for the past two years. 

An examination of past medical records and discussion with his mother provided further history and background. The patient is of Brazilian and European descent and split his childhood between Brazil and the United States. Prior to his diagnosis of Niemann–Pick disease, he was healthy, employed, and able to perform daily activities without any difficulties. As a child, he suffered from dyslexia and ADHD, but graduated college with the help of private tutoring and individualized teaching. At age 34, he began experiencing difficulties moving his eyes. This was followed by a sharp cognitive decline that forced him to move in with his parents. He was unable to live independently at that time or complete his activities of daily living. Genetic testing at age 35 identified point mutations at exon 20 (denoted at the DNA level as c.2974G>T and at the protein level as p.G992R) and 21 (denoted at the DNA level as c.3104C>T and at the protein level as p.A1035V) of the *NPC1* gene. A genomic hybridization microarray was also conducted, demonstrating equivocal results of an interstitial duplication at 12p13.33 spanning approximately 155 kb. Parental testing was not performed.

Since his diagnosis, the patient’s condition progressively deteriorated. He stopped driving at age 40. At age 44, he became wheelchair-bound. Over the five years prior to his hospital presentation, medical records documented the patient having tremor, alogia, dysphagia, amnesia, ataxia, and difficulty with fine finger movements when examined in outpatient clinics. Abdominal ultrasound performed as part of the initial workup revealed a moderately enlarged spleen measuring 18 cm in length. Family history was not significant for Niemann–Pick or other lysosomal storage disorders. The patient’s mother is 78 years old and in fairly good health. His father is living at 89 years old with diabetes and memory loss. The patient’s sister is 54 years old and in good health. The mother reported memory difficulties from a young age, having to write things down and needing to concentrate harder.

## 3. Discussion

Slowed vertical saccades constitute one of the initial, most common, or sometimes only symptom of NP-C1 in adults [6]. VSGP presents initially with a slowing of vertical saccades but progresses to complete loss, particularly in the downward direction. These findings can be identified at the bedside with the use of an optokinetic tape or an optokinetic drum [6]. Reflexive saccades should be assessed to differentiate between VSGP and vertical ocular motor apraxia (OMA) as both eye movement abnormalities involve impaired vertical saccades [7]. Patients with VSGP will present with abnormal reflexive saccades as well as reduced saccadic range and velocity. The “round the house” sign is seen when patients compensate for a loss of vertical saccades by stringing together oblique saccades when attempting vertical gaze, creating a curved upward movement [8]. The “round the house” sign has also been reported in PSP [9]. 

A combination of adult-onset appendicular or gait ataxia, cognitive decline, dystonia, psychosis, and/or VSGP is suggestive of adult-onset NP-C1, typically presenting in the fourth decade of life [2]. A high level of clinical suspicion is necessary in cases of VSGP, “round the house” sign, and other eye movement abnormalities that are classically suggestive of PSP. Similarly to NP-C1, PSP presents with cognitive impairment, ataxia, and movement disorders. PSP can also affect speech, swallowing, behavior, and mood in affected patients. Eight different subtypes of PSP have been identified, with the extent of abnormal tau protein aggregation and neuronal loss in different regions of the brain representative of each of the eight phenotypes [10,11]. The etiology of PSP is unknown but has been attributed to advanced age and exposure to toxins [12,13,14]. Other diagnoses to consider with VSGP include multiple system atrophy (MSA), spinocerebellar ataxia, Wilson disease, Tay–Sachs disease, Lewy body dementia, Creutzfeldt–Jakob disease, and Huntington’s disease. However, these conditions pose less of a diagnostic dilemma since VSCG appears later in their disease course, allowing other identifying clinical features to appear.

It is unknown what percentage of adult-onset NP-C1 patients present with ADHD in childhood. It may reflect the earliest onset of the toxic effects of this genetic defect. Further studies may explore this incidence. The presence of a treatment option may make screening for NP-C1 cost-effective in all ADHD children, given the enormous costs of care for a disabled adult. Further studies should determine the relationship between learning difficulties and ADHD in childhood and its relationship to the development of adult-onset NP-C1.

A definitive diagnosis of NP-C can be achieved through genetic testing. As mentioned above, the patient had point mutations at exon 20 of the *NPC1* gene, a pathogenic variant reported in the literature [15]. If genetic testing results are negative or inconclusive, the filipin test is used. The filipin test was the first diagnostic test for NP-C and was considered the gold standard for NP-C diagnosis until it was recently superseded by genetic testing due to its invasive nature and high cost. It is positive in 80–85% of NP-C cases [16]. The filipin staining test visualizes the accumulation of unesterified cholesterol in the late endosomal–lysosomal pathway via fluorescence microscopy after staining cultured skin fibroblasts with filipin, a fluorescent compound isolated from *Streptomyces filipinesis* [17]. Other abnormal biomarkers seen in NP-C include oxysterol. Elevations in plasma cholestane-3β,5α,6β-triol (C-triol) and 7-ketocholesterol are observed. C-triol is the preferred oxysterol biomarker because of its higher sensitivity and specificity for NP-C [18]. MRI findings are nondiagnostic alone but may reveal cerebral, cerebellar, or midbrain atrophy and white matter hyperintensities [19]. The patient in the current case also demonstrated equivocal results on genomic hybridization microarray. The interstitial duplication at 12p13.33 contains a portion of the CACNA1C gene. The importance of this finding is unclear as mutations of this gene are associated with Timothy syndrome [20]. Neither the patient’s clinical presentation nor their genomic hybridization microarray findings are consistent with Timothy syndrome.

The NP-C Suspicion Index (SI) is a screening tool developed to facilitate diagnosis and early detection, especially for physicians who are unfamiliar with the disorder [4]. The SI tool incorporates several components of the patient’s clinical picture, assessing neurological, psychiatric, and visceral manifestations to establish a Risk Prediction Score (RPS). The SI tool recommends immediate testing if patients achieve an RPS at or above 70 and suggests a low probability of NP-C at RPSs below 40. An investigation by Wraith et al. analyzed the SI tool, concluding that it is not useful for patients younger than 4 years but maintains strong discriminatory power in patients older than 4 years [21]. With respect to adult-onset NP-C, patients likely fall in a score range between 40 and 70. This highlights the diagnostic challenge and delay seen clinically in patients with NP-C, with reports of diagnostic delays averaging at 13.5 years [19,22,23]. These delays can be especially detrimental as treatment initiation at the time of symptom onset is the most important outcome parameter for neurological symptom management [24]. Additional studies are needed to determine its utility for late adult-onset NP-C.

Sea-blue histiocytes are ceroid-laden atypical macrophages that turn blue with May–Giemsa staining. They represent another finding in NP-C and are thought to develop from the ceroid transformation of accumulated sphingomyelin in histiocytes [25]. Sea-blue histiocytes can represent a rare primary disease, or a secondary finding in other conditions. Along with foam cells, they can be seen in diseases with high cell turnover, thrombocytopenia, and splenomegaly such as leukemia, polycythemia vera, and myelodysplastic syndrome, and in lipid and lysosomal disorders, such as NP-C and Gaucher disease [26,27]. 

Miglustat (N-butyldeoxynojirimycin) is the first-choice treatment option for patients with NP-C [28]. It should be started early but, as in our case, does not arrest the neurological decline. Positive outcomes with this treatment have been demonstrated in a comprehensive registry of 18 adolescent and adult-onset NP-C patients treated with miglustat [29]. However, a previous case highlighted the concern for tolerance by patients. Piroth et al. described a patient diagnosed with NP-C at age 34 with an initial symptom of cognitive decline at 17 years and ataxia at 23 years [30]. The patient had received 300mg/day of miglustat for more than 2.5 years following diagnosis but continued to experience worsening symptoms. A dose escalation of 600 mg/day was attempted twice for this patient but was not successful as the patient experienced worsening ataxia and developed a tremor. This specific case demonstrates both the importance of early initiation and medication intolerance at higher doses in severely affected patients. A reversal of intracellular accumulations of unesterified cholesterol in neuronal cell lines occurred in clinical trials using 2-hydroxypropyl-β-cyclodextrin. Another potential new medication is N-acetyl-L-leucine for Niemann–Pick type C. This medication is currently under a multinational double-blind randomized control study [31]. Studies have also shown histone deacetylase (HDAC) inhibitors, such as vorinostat, can reduce cholesterol accumulation in fibroblasts derived from *NPC1* patients. While vorinostat is approved for the treatment of cutaneous T-cell lymphoma, further studies are pending to study its use in NP-C.

## 4. Conclusions

NP-C1 should be considered in the differential diagnosis of patients presenting with new-onset slow vertical saccades and VSGP. This may accompany or develop into slow progressive ataxia, gait impairment, and visuo-executive cognitive impairment. It remains unclear if learning difficulties in adolescents are associated with adult-onset NP-C1. The relationship of learning disability to the clinical appearance of NP-C1 should be investigated to see if earlier diagnosis with screening is cost-effective. Plasma biomarkers along with genetic testing are currently the diagnostic standard for adult-onset NP-C1. Elevations in C-triol and 7-ketocholesterol are useful biomarker findings seen in NP-C. MRI findings are non-specific but may include cerebral, cerebellar, or midbrain atrophy and white matter hyperintensities. The NP-C Suspicion Index screening tool may be beneficial in establishing a diagnosis, but further studies are needed to determine its importance in adult-onset NP-C1. While there is no cure for NP-C, treatment focuses on symptomatic management and slowing disease progression. Miglustat, a glucosylceramide synthase inhibitor, is the first disease-specific therapy that has been used to slow and prevent the neurological manifestations of NP-C. Improved outcomes in terms of minimizing neurological symptoms can be established by reducing time between initial symptom presentation and treatment initiation. In patients with delayed treatment initiation and thus a need for higher dosage for symptomatic management, tolerance and adverse events may be a concern. Future studies should investigate the relationship between dose tolerance, treatment initiation, and treatment efficacy.

## Data Availability

Data are contained within the article.

## Supplementary Materials

The following supporting information can be downloaded at: https://www.mdpi.com/article/10.3390/neurolint16030042/s1, Video S1: Supranuclear palsy in a patient diagnosed with adult-onset Niemann–Pick type C.
